# Antidiabetic Activity of *Acacia tortilis* (Forsk.) Hayne ssp. raddiana Polysaccharide on Streptozotocin-Nicotinamide Induced Diabetic Rats

**DOI:** 10.1155/2014/572013

**Published:** 2014-07-09

**Authors:** Pradeep Kumar Bhateja, Randhir Singh

**Affiliations:** ^1^Department of Pharmacology, Bhojia Dental College and Hospitals, Bhud, Baddi, Himachal Pradesh 173205, India; ^2^Department of Pharmacology, M. M. University, Mullana, Ambala, Haryana 133207, India

## Abstract

The present study was designed to investigate the antidiabetic activity of aqueous extract of *Acacia tortilis* polysaccharide (AEATP) from gum exudates and its role in comorbidities associated with diabetes in STZ-nicotinamide induced diabetic rats. Male albino Wistar rats were divided into control, diabetic control, glimepiride treated (10 mg/kg), and diabetic rats treated with 250, 500, and 1000 mg/kg dose of AEATP groups and fasting blood glucose, glycated hemoglobin, total cholesterol, triglyceride, LDL, VLDL, HDL, SGOT, and SGPT levels were measured. STZ significantly increased fasting blood glucose level, glycated hemoglobin, total cholesterol, triglyceride, LDL, VLDL, SGOT, and SGPT levels, whereas HDL level was reduced as compared to control group. After 7 days of administration, 500 and 1000 mg/kg dose of AEATP showed significant reduction (*P* < 0.05) in fasting blood glucose level compared to diabetic control. AEATP has also reduced total cholesterol, triglyceride, LDL, VLDL, SGOT, and SGPT levels and improved HDL level as compared to diabetic control group. Our study is the first to report the normalization of fasting blood glucose level, lipid profile, and liver enzyme in AEATP treated diabetic rats. Thus, it can be concluded that AEATP may have potentials for the treatment of T2DM and its comorbidities.

## 1. Introduction

Diabetes mellitus (DM) is characterized by hyperglycemia and is associated with a group of metabolic disorders, that is, abnormalities in carbohydrate, fat and protein metabolism which further result in chronic complications including microvascular, macrovascular, and neuropathic disorder [1]. It is dispersed worldwide with prevalence from 171 million in 2000 to 366 million in 2030 [2].

The currently available oral hypoglycemic and antihyperglycemic drugs for type-II diabetes have their own limitations, adverse effects, and secondary failures. Therefore, to reduce their cost, limitation, and adverse effects, focus has been shifted towards the medicinal herbs for safe and effective use. Recently a lot of medicinal herbs are being investigated for their role in pharmacotherapy of diabetes [3].

Israeli babool and umbrella thorn are the other names of* Acacia tortilis *and are widespread distributed around the globe (Africa, Algeria, Egypt, Asia, Israel, Somalia, Pakistan, and India). In India, this tree was introduced in 1958 from Israel [4]. Various extracts of this plant have the following actions like smooth muscle relaxing activity [5], effective in treatment of *α*_2_-adrenoceptor related diseases [6], antimicrobial activity against* Bacillus subtilis*,* Escherichia coli*,* Staphylococcus aureus*,* Pseudomonas aeruginosa*, and* Candida albicans* [7], in-vitro antiplasmodial and antileishmanial activity [8], antiviral effect against human immunodeficiency virus-1 [9], antiasthmatic [10], and hypotensive and diuretic property [11].

Moreover, various species of Acacia are reported to have antidiabetic activity like* Acacia arabica*,* Acacia catechu*,* Acacia mollissima*,* Acacia polyacantha*, and so forth [12–15]. Previously numerous studies on polysaccharides from* Phellinus linteus* [16], Ascophyllum [17], Taxus [18], Acanthopanax [19], and Andrographis [20] demonstrated the antidiabetic activity. Further, the seed extract of* Acacia tortilis* has been also found to have an antihyperglycemic activity [21].

Thus, with the same line of research, the present study was designed to explore the antidiabetic activity of aqueous extract of* Acacia tortilis* (Forsk.) Hayne ssp. raddiana polysaccharide from gum exudates.

## 2. Material and Methods

### 2.1. Chemicals

Streptozotocin, Glimepiride, and Nicotinamide were procured form Sigma-Aldrich, Milwaukee, USA, and all the other chemicals were of analytical grade.

### 2.2. Plant Material

Gum exudates from the stem and branches of* Acacia tortilis* was collected from Central Arid Zone Research Institute Campus, Jodhpur, India.

### 2.3. Animals

Male albino Wistar rats (150–200 gm) were used in this study and experimental protocol was approved by Institutional Animal Ethics Committee. Animals were kept as per the guidelines of the Committee for the Purpose of Control and Supervision of Experiments on Animals (CPCSEA), Ministry of Environment and Forest, Government of India (Chitkara College of Pharmacy Animal Facility Registration number: 1181/ab/08/CPCSEA). Animals were fed normal chow diet and* ad libitum* under controlled environmental condition of temperature (24–28°C), relative humidity 60–70%, and natural light/dark cycle (12 : 12) and maintained on standard food pellets and tap water* ad libitum*.

### 2.4. Acute Oral Toxicity Studies

Acute oral toxicity studies were performed according to OECD (Organization for Economic Cooperation and Development) 423 guidelines at the dose of 300, 2,000, and 5,000 mg/kg. The general behavior was observed continuously for 48 hr, 3 days and mortality was observed for 14 days [22].

### 2.5. Isolation of Polysaccharide

Gum exudate was crushed into fine particles using laboratory grinder. Fine powder of gum exudates (100 gm) was stirred vigorously in distilled water (200 mL) for 6 hours at room temperature and centrifuged to remove water-insoluble part. The supernatant solution was decanted off. The concentrated aqueous solution was poured into 3 times its volume of ethanol with constant stirring. The polysaccharide was precipitated out in the form of a fluffy precipitate. The precipitate was again dissolved in water and added to ethanol. Precipitate was treated successively with dry solvent ether and acetone. It was filtered under vacuum and dried in vacuum desiccators at room temperature [23].

### 2.6. Complete Hydrolysis

The pure polysaccharide was subjected to hydrolysis with sulfuric acid (2N) for 18 hr on steam bath. The hydrolyzate was cooled, neutralized with saturated solution of barium carbonate by dropwise addition till the pH of the solution reached at 7, filtered, and the residue washed with water. The combined filtrate was concentrated at or below 40°C in rotary evaporator under reduced pressure. This hydrolyzed mass was used for paper chromatography.

### 2.7. Experimental Protocol

In experimental animals, diabetes was induced by intraperitoneal injection of Nicotinamide (230 mg/kg) 15 min before streptozotocin (65 mg/kg, i.p) administration [24]. The STZ was freshly prepared by dissolving in 0.1 M citrate buffer, pH 4.5, and nicotinamide was prepared in normal saline. Diabetes mellitus was confirmed after 14 days of STZ administration when fasting blood glucose level had become constant above 250 mg/dL. As STZ is capable of inducing fatal hypoglycemia as a result of massive pancreatic insulin release, STZ-treated rats were provided with 10% glucose solution after 6 hr for the next 24 hr to prevent fatal hypoglycemia [25]. Different doses of AEATP (250, 500, and 1000 mg/kg) were administered to the animals and doses were selected on the basis of acute toxicity studies. [26].

Further, animals with blood glucose level above 250 mg/dL were selected and divided into six groups comprising ten animals in each group.

 Group 1: control: no intervention.
 Group 2: diabetic control + vehicle distilled water.
 Group 3: diabetic rats + glimepiride (10 mg/kg body weight).
 Group 4: diabetic rats + 250 mg/kg AEATP.
 Group 5: diabetic rats + 500 mg/kg AEATP.
 Group 6: diabetic rats + 1000 mg/kg AEATP.

After 29 days of continuous treatment, blood sample was collected from retroorbital plexus under anesthesia for biochemical estimation of total cholesterol, triglycerides, LDL, HDL, VLDL, HbA1c, SGOT, and SGPT by commercially available kits of Reckon Diagnostics Pvt., Ltd. Body weight was measured before induction of diabetes and during treatment period. Fasting blood glucose level of overnight fasted rats was measured using glucometer on the 1st, 7th, 14th, 21st, and 28th days of pharmacological interventions. Serum insulin was measured by using ELISA kits (EMD Millipore-EZRMI-13 K). The assay is based on Sandwich ELISA technique and enzyme activity was measured spectrophotometrically by the increased absorbency at 450 nm, corrected from the absorbency at 590 nm, after acidification of formed products. Pancreatic insulin content was extracted by taking 0.2 g pancreas portion with 5.0 mL of ice-cold acid-alcohol in a centrifuge tube, homogenized, followed by sonication, stored at −20°C overnight, and finally centrifuged at 3000 rpm at 4°C for 15 minutes. The supernatant was transferred into a new centrifuge tube and stored at −20°C, while the pellet was subjected to extraction again. After extraction insulin level was measured at room temperature by ELISA assay kit [27].

### 2.8. Statistical Analysis

Statistical analysis was performed using Graph pad Prism 6. Values are expressed as mean ± SEM and statistical analysis was carried out by using by ANOVA followed by Tukey's as* post hoc* multiple comparison test.

## 3. Results

The pure polysaccharide was extracted as amorphous white powder with a percentage yield of 24.5% from the gum exudates. Complete hydrolysis of the polysaccharide followed by paper chromatography revealed the presence of four spots, corresponding to D-galactose, D-glucose, L-rhamnose, and D-glucuronic acids, respectively.

### 3.1. Acute Oral Toxicity Studies

In the present study, oral toxicity was carried out according to OECD guidelines, up to an elevated concentration of 5,000 mg/kg. However, at this dose* Acacia tortilis* polysaccharide did not exhibit any sign of toxicity, behavioral changes, and mortality. Thus* Acacia tortilis* polysaccharide was found to be nontoxic and therefore can be safely used.

### 3.2. Effect of AEATP on Body Weight

**Figure 1:**
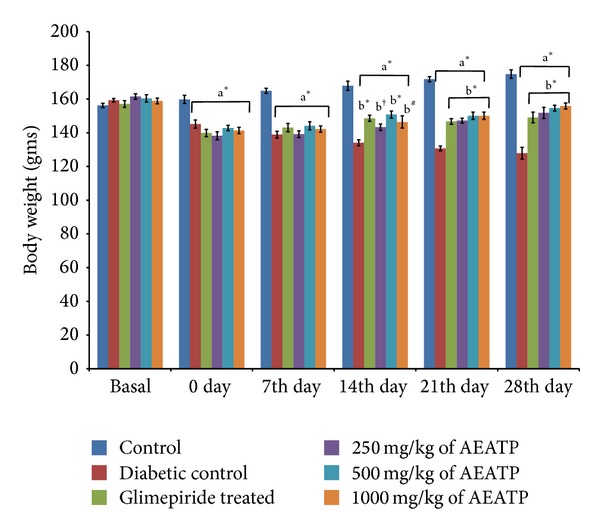
Effect of AEATP on body weight (gms) in type-2 diabetic Wistar rats. Each group (*n* = 6) represents mean ± standard error of means. Data was analyzed by using Two way ANOVA followed by Tukey's multiple test; a versus control, b versus Diabetic control, c versus Glimepiride treated, d versus 250 mg/kg of AEATP, e versus 500 mg/kg of AEATP. *P < 0.0001, ^#^ *P* < 0.001, ^†^ *P* < 0.01, ^‡^ *P* < 0.05.

Animals of the same weight range were used in experimental protocol. During the study, the body weight of control group was increased naturally, whereas body weight was found to be significantly attenuated in STZ-induced diabetic group as compared to control group (Figure 1). After 28 days of continuous administration of glimepiride, a significant increase in body weight was observed as compared to diabetic control group. Oral administration of AEATP to diabetic rats also significantly increased the body weight at the 14th day of intervention and reversed the effect of STZ comparable to glimepiride treated group. Furthermore, on 21st and 28th days of treatment, no significant difference in body weight was observed between glimepiride, 250, 500, and 1000 mg/kg of AEATP treated groups and the effect of AEATP administration produced dose-independent effect on body weight in diabetic rats.

### 3.3. Effect of AEATP on Fasting Blood Glucose Level

**Figure 2:**
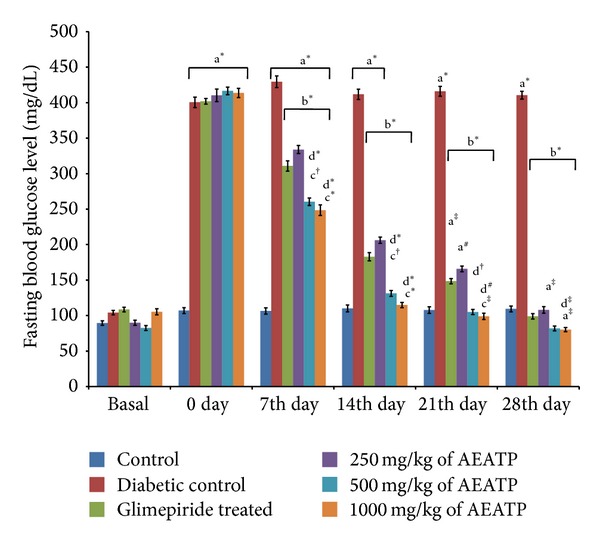
Effect of AEATP on fasting blood glucose level (mg/dL) in type-2 diabetic Wistar rats. Each group (*n* = 6) represents mean ± standard error of means. Data was analyzed by using Two way ANOVA followed by Tukey's multiple test; a versus control, b versus Diabetic control, c versus Glimepiride treated, d versus 250 mg/kg of AEATP, e versus 500 mg/kg of AEATP. *P < 0.0001, ^#^ *P* < 0.001, ^†^ *P* < 0.01, ^‡^ *P* < 0.05.

The basal values of fasting blood glucose level were almost the same and statistically no significant difference was observed while including the animals for experimentation. Fasting blood glucose level of control group ranged from 106.5 ± 6.64 to 110.16 ± 3.33 mg/dL in 28 days of study, while there was a significant increase in fasting blood glucose level in STZ + nicotinamide treated rat 400.5 ± 7.8 mg/dL as compared to control group (Figure 2). Fasting blood glucose level glucose level was measured on the 7th, 14th, 21st, and 28th days. Different doses of AEATP (250, 500, and 1000 mg/kg) were administered continuously for 28 days and significant reduction in blood glucose level was observed in the STZ + NAD treated diabetic rats. Similarly, glimepiride significantly reduced fasting blood glucose level measured on the 7th, 14th, 21st, and 28th days. However, hypoglycemia was not observed even on the 28th day of continuous administration of AEATP since as per CPCSEA normal blood glucose range of Wistar albino rat is 50–135 mg/dL. Interestingly, on the 7th and 14th days, both 500 and 1000 mg/kg of AEATP and the 21st day only 1000 mg/kg of AEATP also showed significant reduction in blood glucose level compared to glimepiride and 250 mg/kg of AEATP, whereas, on the 28th day, only 1000 but not 500 mg/kg of AEATP showed significant reduction in blood glucose level compared to 250 mg/kg of AEATP.

### 3.4. Effect of AEATP on Glycated Hemoglobin (HbA1c)

**Figure 3:**
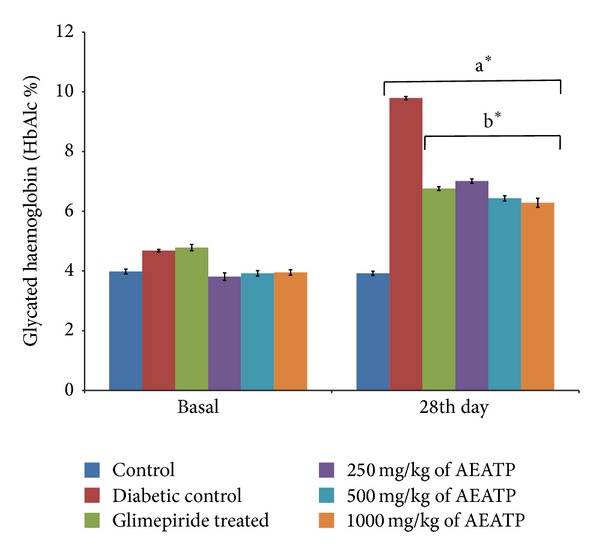
Effect of AEATP on glycated hemoglobin (HbA1c) in type-2 diabetic Wistar rats. Each group (*n* = 6) represents mean ± standard error of means. Data was analyzed by using Two way ANOVA followed by Tukey's multiple test; a versus control, b versus Diabetic control, c versus Glimepiride treated, d versus 250 mg/kg of AEATP, e versus 500 mg/kg of AEATP. *P < 0.0001, ^#^ *P* < 0.001, ^†^ *P* < 0.01, ^‡^ *P* < 0.05.

Glycated hemoglobin was significantly increased in STZ-induced diabetic group 9.79 ± 0.2% as compared to control group 3.92 ± 0.19% (Figure 3). After 28 days of treatment, with 250, 500, and 1000 mg/kg of AEATP and glimepiride shown significant attenuation in elevated glycated hemoglobin level as compared to diabetic control group, that is, 7.01 ± 0.12%, 6.43 ± 0.09%, 6.28 ± 0.13%, and 6.76 ± 0.11, respectively.

### 3.5. Effect of AEATP on Total Cholesterol Level

**Figure 4:**
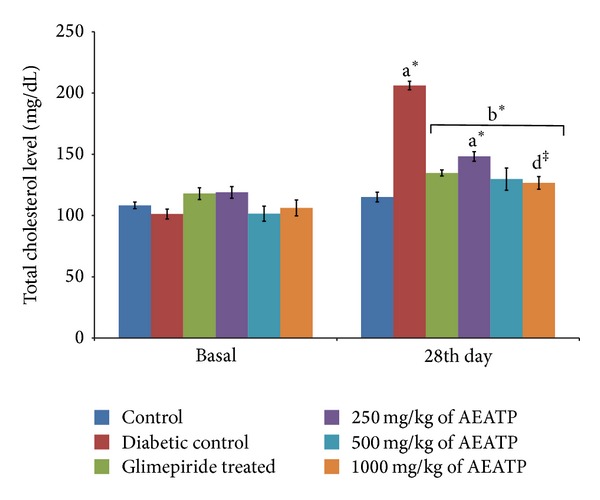
Effect of AEATP on total cholesterol (mg/dL) in type-2 diabetic Wistar rats. Each group (*n* = 6) represents mean ± standard error of means. Data was analyzed by using Two way ANOVA followed by Tukey's multiple test; a versus control, b versus Diabetic control, c versus Glimepiride treated, d versus 250 mg/kg of AEATP, e versus 500 mg/kg of AEATP. *P < 0.0001, ^#^ *P* < 0.001, ^†^ *P* < 0.01, ^‡^ *P* < 0.05.

The present result showed that total cholesterol level in serum was significantly elevated in diabetic control group 206.1 ± 3.7 mg/dL as compared to control group 115.1 ± 3.1 mg/dL (Figure 4). Different doses of (250, 500, and 1000 mg/kg) of AEATP produced statistically significant reduction in cholesterol level 148.1 ± 4.58 mg/dL, 129.6 ± 2.55 mg/dL, and 126.0 ± 6.51 mg/dL, respectively, as compared to diabetic control group after 28 days of treatment. 500 and 1000 mg/kg of AEATP except 250 mg/kg brought down the elevated cholesterol level to normal and also significantly reduced the total cholesterol level comparable to glimepiride treated group, that is, 134.2 ± 3.52 mg/dL.

### 3.6. Effect of AEATP on Total Triglycerides, LDL, and VLDL Levels

**Figure 5:**
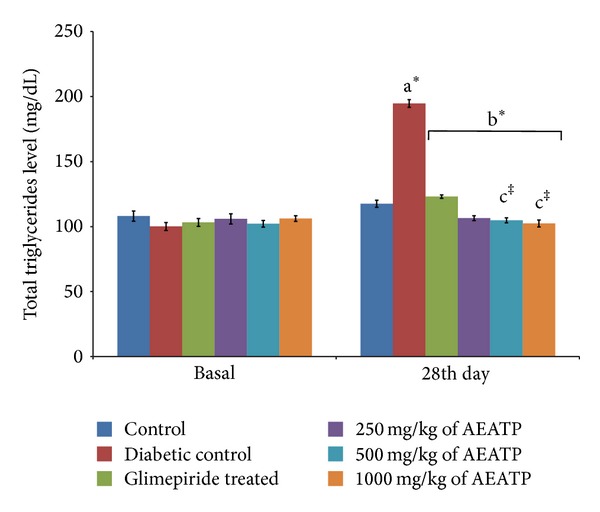
Effect of AEATP on total triglyceride level (mg/dL) in type-2 diabetic Wistar rats. Each group (*n* = 6) represents mean ± standard error of means. Data was analyzed by using Two way ANOVA followed by Tukey's multiple test; a versus control, b versus Diabetic control, c versus Glimepiride treated, d versus 250 mg/kg of AEATP, e versus 500 mg/kg of AEATP. *P < 0.0001, ^#^ *P* < 0.001, ^†^ *P* < 0.01, ^‡^ *P* < 0.05.

**Figure 6:**
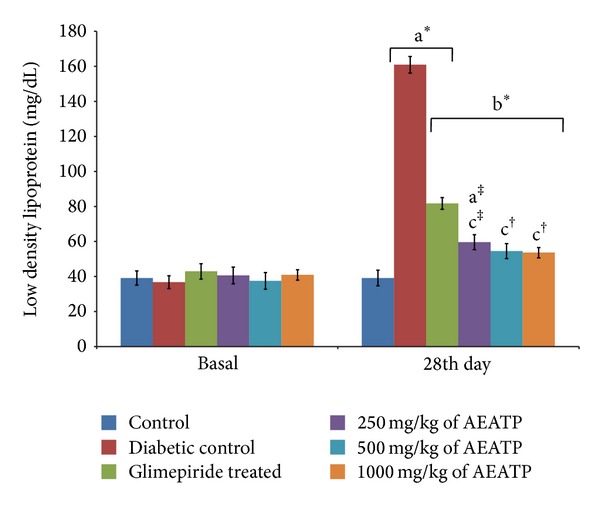
Effect of AEATP on low density lipoprotein (LDL) level (mg/dL) in type-2 diabetic Wistar rats. Each group (*n* = 6) represents mean ± standard error of means. Data was analyzed by using Two way ANOVA followed by Tukey's multiple test; a versus control, b versus Diabetic control, c versus Glimepiride treated, d versus 250 mg/kg of AEATP, e versus 500 mg/kg of AEATP. *P < 0.0001, ^#^ *P* < 0.001, ^†^ *P* < 0.01, ^‡^ *P* < 0.05.

**Figure 7:**
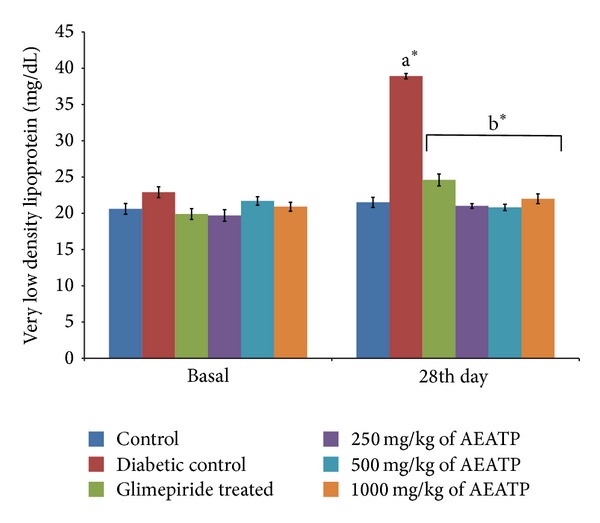
Effect of AEATP on very low density lipoprotein (VLDL) level (mg/dL) in type-2 diabetic Wistar rats. Each group (*n* = 6) represents mean ± standard error of means. Data was analyzed by using Two way ANOVA followed by Tukey's multiple test; a versus control, b versus Diabetic control, c versus Glimepiride treated, d versus 250 mg/kg of AEATP, e versus 500 mg/kg of AEATP. *P < 0.0001, ^#^ *P* < 0.001, ^†^ *P* < 0.01, ^‡^ *P* < 0.05.

There was significant increase in total triglyceride (TG), low density lipoprotein (LDL), and very low density lipoprotein (VLDL) levels in diabetic control group when compared to control group. The administration of AEATP and glimepiride for 28 days significantly attenuated diabetes induced high level of TG, LDL, and VLDL. 250, 500, and 1000 mg/kg AEATP and glimepiride significantly reduced the TG level by 106.73 ± 2.6, 104.87 ± 2.0, 102.3 ± 3.7, and 123.14 ± 4.8 mg/dL, respectively, as compared to diabetic control group 194.67 ± 3.41 mg/dL (Figure 5). Similarly, significant attenuation was observed in LDL level with 250, 500, and 1000 mg/kg AEATP and glimepiride treatment, that is, 59.62 ± 5.24, 54.62 ± 5.9, 53.76 ± 3.77, and 81.79 ± 4.17 mg/dL, respectively as compared to diabetic control group 160.97 ± 6.92 mg/dL (Figure 6). Moreover, 250, 500, and 1000 mg/kg AEATP and glimepiride significantly reversed the VLDL level, that is, 21.01 ± 0.28, 20.98 ± 0.4, 22.09 ± 0.7, and 24.63 ± 0.96 mg/dL, respectively as compared to diabetic control 38.93 ± 0.68 mg/dL (Figure 7).

### 3.7. Effect of AEATP on High Density Lipoprotein Level (HDL)

**Figure 8:**
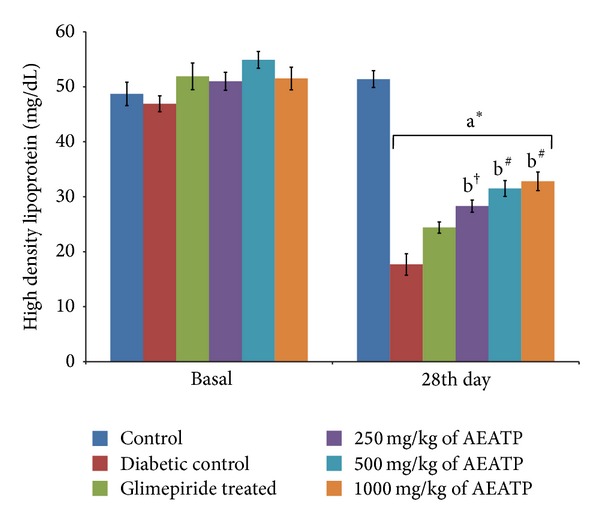
Effect of AEATP on high density lipoprotein (HDL) level (mg/dL) in type-2 diabetic Wistar rats. Each group (*n* = 6) represents mean ± standard error of means. Data was analyzed by using Two way ANOVA followed by Tukey's multiple test; a versus control, b versus Diabetic control, c versus Glimepiride treated, d versus 250 mg/kg of AEATP, e versus 500 mg/kg of AEATP. *P < 0.0001, ^#^ *P* < 0.001, ^†^ *P* < 0.01, ^‡^ *P* < 0.05.

HDL level was found to be significantly reduced in diabetic control group 17.75 ± 2.73 mg/dL compared to control group 51.43 ± 2.95 mg/dL. After 28 days of intervention with 250, 500, and 1000 mg/kg of AEATP, a significant increase was observed in HDL level 28.35 ± 1.28 mg/dL, 31.55 ± 2.79 mg/dL, and 32.87 ± 3.3 mg/dL, respectively, as compared to diabetic control group 17.75 ± 2.73 mg/dL. Administration of glimepiride also increased HDL level of diabetic rats 24.44 ± 1.3 (Figure 8).

### 3.8. Effect of AEATP on Liver Enzymes (unit/lit.)

**Figure 9:**
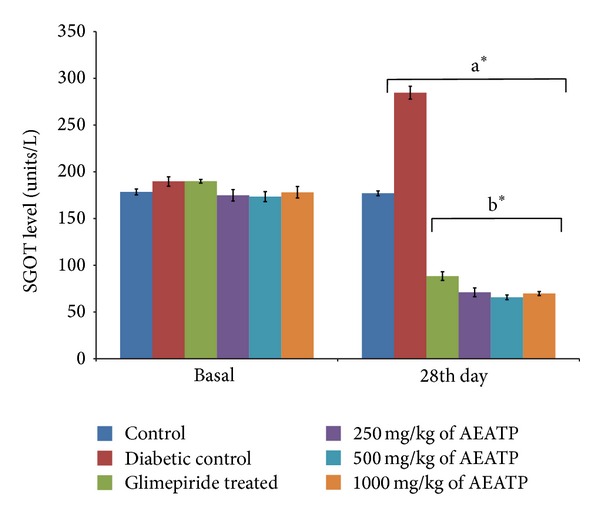
Effect of AEATP on SGOT level (unit/lit.) in type-2 diabetic Wistar rats. Each group (*n* = 6) represents mean ± standard error of means. Data was analyzed by using Two way ANOVA followed by Tukey's multiple test; a versus control, b versus Diabetic control, c versus Glimepiride treated, d versus 250 mg/kg of AEATP, e versus 500 mg/kg of AEATP. *P < 0.0001, ^#^ *P* < 0.001, ^†^ *P* < 0.01, ^‡^ *P* < 0.05.

**Figure 10:**
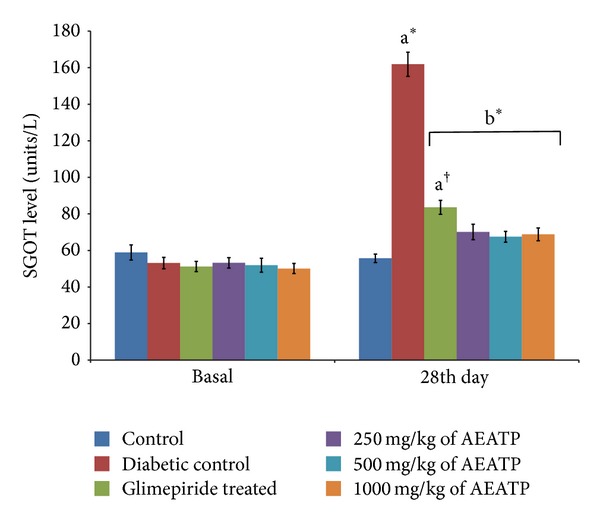
Effect of AEATP on SGPT level (unit/lit.) in type-2 diabetic Wistar rats. Each group (*n* = 6) represents mean ± standard error of means. Data was analyzed by using Two way ANOVA followed by Tukey's multiple test; a versus control, b versus Diabetic control, c versus Glimepiride treated, d versus 250 mg/kg of AEATP, e versus 500 mg/kg of AEATP. *P < 0.0001, ^#^ *P* < 0.001, ^†^ *P* < 0.01, ^‡^ *P* < 0.05.

A significant elevation in SGOT and SGPT was observed in serum of STZ-induced diabetic rats 284.5 ± 10.21 and 161.9 ± 5.21 unit/lit. as compared to control group 177.0 ± 2.4 and 55.76 ± 3.09 unit/lit., respectively. A statistically significant attenuation was observed in SGOT enzyme level after 28 days of administration of 250, 500, and 1000 mg/kg of AEATP (71.16 ± 6.79 and 65.76 ± 4.31 unit/lit. 69.87 ± 3.61, resp.) and glimepiride (88.50 ± 5.22 unit/lit.) as compared to diabetic control 284.5 ± 10.21 unit/lit. (Figure 9). Similar results were observed in SGPT enzyme level with 250, 500, and 1000 mg/kg of AEATP and glimepiride, that is, 70.11 ± 6.75, 67.53 ± 5.70, 68.87 ± 4.10, and 83.63 ± 4.92 unit/lit., respectively, when compared to diabetic control group 161.9 ± 5.21 unit/lit. (Figure 10).

### 3.9. Effect of AEATP on Fasting Insulin Level and Pancreatic Insulin Content

A significant decrease was observed in fasting insulin level (0.373 ± 0.026 ng/mL) and pancreatic insulin content (56.0 ± 2.81 ng/mg pancreas) in STZ-induced diabetic rats as compared to control group 0.820 ± 0.024 ng/mL and 103.9 ± 5.24 ng/mg pancreas, respectively. Fasting insulin level was statistically increased after 28 days administration of 250, 500, and 1000 mg/kg of AEATP (0.550 ± 0.024, 0.613 ± 0.020, and 0.683 ± 0.024 ng/mL, resp.) and glimepiride (0.723 ± 0.024 ng/mL) as compared to diabetic control (0.373 ± 0.026 ng/mL) (Table 1). Similar results were observed in pancreatic insulin content with 250, 500, and 1000 mg/kg of AEATP and glimepiride (67.0 ± 2.98, 77.33 ± 5.68, 92.46 ± 3.14, and 99.9 ± 2.22 ng/mg pancreas, resp.) when compared to diabetic control group (56.0 ± 2.81 ng/mg pancreas) (Table 2).

## 4. Discussion

The present study was designed to investigate antidiabetic activity of AEATP along with its effect on lipid profile and liver enzymes. STZ (N-nitro derivative of glucosamine) has potent alkylating property [28] and is specifically cytotoxic to the pancreatic beta cells in mammals. The pancreatic beta cell preferentially uptakes STZ resulting in formation of superoxide radicals. Moreover, NO moiety is liberated from STZ leading to the destruction of *β*-cells by necrosis [25]. Recently, a new animal model of type 2 diabetes has been introduced in which a combination of STZ and nicotinamide administration is able to induce DM in adult rats. The rats administered nicotinamide (230 mg/kg, ip) 15 min before STZ (65 mg/kg, ip) was found to develop moderate and stable nonfasting hyperglycaemia without any significant change in plasma insulin level. Nicotinamide is an antioxidant which exerts protective effect on the cytotoxic action of STZ by scavenging free radicals and causes only minor damage to pancreatic *β*-cell mass producing type 2 diabetes [24]. Like previous reports, significant increase in fasting blood glucose level was observed in STZ induced diabetic rats compared to control group [29, 30]. Administration of AEATP for 28 days resulted in significant reduction in the fasting blood glucose level as compared to diabetic rats. It is evident from this investigation that the aqueous extract was effective in maintaining the blood glucose levels in STZ and nicotinamide induced diabetic rats. Interestingly, on the 7th and 14th days, both 500 and 1000 mg/kg of AEATP and the 21st day only 1000 mg/kg of AEATP also showed significant reduction in blood glucose level as compared to diabetic control and 250 mg/kg of AEATP, whereas on the 28th day, only 1000 but not 500 mg/kg of AEATP showed significant reduction in blood glucose level as compared to 250 mg/kg of AEATP.

Induction of diabetes is associated with the characteristic loss of body weight [31, 32], which is due to muscle wasting and catabolism of tissue proteins leading to significant reduction in the body weight in diabetic rats. Similar effect was also observed in the present study. Diabetic rats treated with AEATP, independent of dose, showed an increase in the body weight as compared to the diabetic control which might be due to its protective effect in controlling muscle wasting, that is, reversal of gluconeogenesis, and might also be due to the improvement in insulin secretion and glycemic control.

Glycated hemoglobin (HbA1c) is the standard biochemical marker in assessment of diabetes. In our study, diabetic rats showed higher level of glycated hemoglobin indicating their poor glycemic control which is also supported by other previously reported studies [33, 34]. Oral administration of AEATP at all the doses significantly reduced HbA1c to near normalcy by 28 days of intervention as compared to diabetic control group. Several studies have demonstrated that flavonoids attenuate hyperglycemia [35] and reduced nonenzymatic glycation of proteins in animals [36] as* Acacia tortilis* was also reported to have flavonoids content [37] and might show the similar activity.

Lipid plays an important role in the pathogenesis of complications associated with diabetes mellitus. The elevated level of serum cholesterol and reduced level of serum HDL cholesterol in diabetic condition, poses to be a risk factor for developing microvascular complication leading to atherosclerosis and cardiovascular diseases like coronary heart disease. The abnormal high concentration of serum lipid in diabetic mainly due to increased mobilization of free fatty acids from peripheral fat depots, since insulin inhibits the hormone sensitive lipase, insulin deficiency, or insulin resistance may be responsible for dislipidemia [38]. The present study showed that diabetic rats has abnormal lipid profile as earlier reports [39, 40], whereas the AEATP treated group showed significant improvement in the lipid profile comparable to glimepiride treated group. Interestingly, 500 and 1000 mg/kg of AEATP have shown significant reduction of TG and LDL-C as compared to glimepiride treated group indicating additional hypolipidemic activity over available standard drugs. Hypolipidemic effect could represent a protective mechanism against the development of atherosclerosis. It is well known that hyperlipidemia has an association with atherosclerosis and the incidence of atherosclerosis is increased in diabetics [41].

There was increase in liver enzymes in serum of diabetic rats like earlier reports [42] which might be primarily due to leakage of these enzymes from liver cytosol into bloodstream as a consequence of hepatotoxic effect of STZ. AEATP, at all the doses, lowered serum SGPT and SGOT levels which showed the protective effect and normal functioning of liver in reversing the organ damage due to diabetes which was clearly observed by high levels of SGOT and SGPT in diabetic control.

The possible mechanism of action of AEATP could be correlated with promoting insulin secretion by closure of K^+^-ATP channels, membrane depolarization, and stimulation of Ca^2+^ influx, an initial key step in insulin secretion. As studies have shown that the gut incretin content is altered in animal models of type 2 diabetes [43], therefore AEATP may act by increasing incretin, that is, increasing glucagon-like-peptide-1 (GLP-1) or inhibiting dipeptidyl peptidase-4 (DPP-4). Furthermore, glucagon-like-peptide-1 reportedly promotes islet cell growth and inhibits apoptosis in animal models; an increase in GLP-1 secretion might also be beneficial for islet cell function and mass in humans [44]. AEATP exerts protective effects in experimental diabetes, possibly by reducing oxidative stress, and hence protects rats from oxidative damage and dyslipidemia due to STZ treatment. However, further studies are necessary to confirm these effects. The results of our study confirm that the AEATP has a potent antidiabetic activity reversing the disturbances in lipid profile and liver toxicity.

This study revealed first time the antidiabetic activity of AEATP and its effective role in maintaining lipid profile and liver toxicity in diabetic rats. This new insight allows an understanding of the use of* Acacia tortilis* polysaccharide in prevention and treatment of diabetes, hyperlipidemia, and its associated complications. However, the precise mechanism by which AEATP reduced fasting blood glucose level in diabetic rats will require further detailed study. Therefore, future research and clinical trials in this area may lead to the use of* Acacia tortilis *polysaccharide as a new type of therapeutic agent in treatment of type 2 diabetes.

## Figures and Tables

**Table 1:** Effect of AEATP on fasting insulin level (ng/mL) in type-2 diabetic Wistar rats.

Fasting insulin level (ng/mL)	Control	Diabetic control	Glimepiride treated	250 mg/kg of AEATP	500 mg/kg of AEATP	1000 mg/kg of AEATP
Basal	0.776 ± 0.026	0.803 ± 0.041	0.783 ± 0.041	0.830 ± 0.035	0.790 ± 0.049	0.803 ± 0.067
28th day	0.820 ± 0.024	0.373 ± 0.026^a#^	0.723 ± 0.024^b#^	0.550 ± 0.024^b#^	0.613 ± 0.020^b#^	0.683 ± 0.024^b#, c‡^

Each group (*n* = 6) represents mean ± standard error of means. Data was analyzed by using one-way ANOVA followed by Tukey's multiple test; a control, b diabetic control, c 500 mg/kg of AEATP. *P < 0.0001, ^#^ *P* < 0.001, ^†^ *P* < 0.01, ^‡^ *P* < 0.05.

**Table 2:** Effect of AEATP on pancreatic insulin content (ng/mg pancreas) in type-2 diabetic Wistar rats.

Pancreatic insulin content (ng/mg pancreas)	Control	Diabetic control	Glimepiride treated	250 mg/kg of AEATP	500 mg/kg of AEATP	1000 mg/kg of AEATP
Basal	106.6 ± 3.35	101.9 ± 2.62	102.3 ± 3.66	101.9 ± 5.75	97.8 ± 5.99	100.9 ± 5.81
28th day	103.9 ± 5.24	56.0 ± 2.81^a#^	99.9 ± 2.22^b#^	67.0 ± 2.98^b‡^	77.33 ± 5.68^b#, c#^	92.46 ± 3.14^b#, c#, d†^

Each group (*n* = 6) represents mean ± standard error of means. Data was analyzed by using one-way ANOVA followed by Tukey's multiple test; a control, b diabetic control, c 250 mg/kg of AEATP, d 500 mg/kg of AEATP. *P < 0.0001, ^#^ *P* < 0.001, ^†^ *P* < 0.01, ^‡^ *P* < 0.05.

## Abbreviations

T2DM: Type 2 diabetes mellitus
HbA1c: Glycated hemoglobin
LDL: Low density lipoprotein
HDL: High density lipoprotein
VLDL: Very low density lipoprotein
S.E.M: Standard error of means
STZ: Streptozotocin
ANOVA: Analysis of variance
AEATP: Aqueous extract of* Acacia tortilis* polysaccharide
DM: Diabetes mellitus.
